# Expression of Selected Epithelial-Mesenchymal Transition Transcription Factors in Endometrial Cancer

**DOI:** 10.1155/2020/4584250

**Published:** 2020-12-29

**Authors:** Paweł Sadłecki, Jakub Jóźwicki, Paulina Antosik, Małgorzata Walentowicz-Sadłecka

**Affiliations:** ^1^Department of Obstetrics and Gynecology, Collegium Medicum in Bydgoszcz, Nicolaus Copernicus University, Torun, Bydgoszcz, Poland; ^2^Department of Clinical Pathomorphology, Collegium Medicum in Bydgoszcz, Nicolaus Copernicus University, Torun, Bydgoszcz, Poland

## Abstract

Endometrial cancer (EC) is the most common gynecologic malignancy in developed countries. The aim of this study was to analyze the expression of SNAIL, SLUG, TWIST1, TWIST2, ZEB1, and ZEB 2 in primary tumor and the correlation with morphological and clinical characteristics of EC. The study included 158 patients with EC after surgical treatments: total hysterectomy and lymphadenectomy. The percentages of EC specimens testing positively for the EMT transcription factors were 84.5% for SNAIL, 92.2% for SLUG, 10.9% for TWIST1, 100% for TWIST2, 89% for ZEB1, and 98% for ZEB2. The expression of SLUG in patients with FIGO stage III or IV, type II EC, myometrial invasion ≥ 50% of the uterine wall thickness, and adnexal involvement and in patients with distant metastases was significantly higher. SLUG and ZEB2 expressions were identified as significant predictors of higher FIGO stages (III or IV) on univariate analysis. The overexpression of SLUG was a significant predictor of more aggressive type II EC, myometrial invasion ≥ 50% of the uterine wall thickness, and distant metastases on both univariate and multivariate analysis. Moreover, the overexpression of SLUG and ZEB2 was shown to be significant predictors of adnexal involvement on univariate analysis. ZEB 2 overexpression was identified in multivariate analysis as another independent predictor associated with a lesser likelihood of type II EC. Both univariate and multivariate analyses demonstrated that SLUG expression was the only predictor of 5-year survival in the study group. The overexpression of SLUG was associated with a significant increase in mortality hazard on univariate analysis and was shown to be a highly significant predictor of death on multivariate analysis. *Conclusions*. Selected proteins of the EMT pathway play a role in endometrial carcinogenesis; SLUG and ZEB2 expressions in the primary tumor might predict clinical outcomes in EC and drive therapeutic decisions regarding adjuvant treatment in patients with this malignancy.

## 1. Introduction

Endometrial cancer (EC) is the most common gynecologic malignancy in developed countries, representing nearly 5% of all female cancers and being responsible for more than 2% of cancer-related deaths in women worldwide [1]. The incidence and mortality of EC have been increasing in recent years and are expected to increase even further [2–5]. In 75% of patients, EC is diagnosed after menopause and in 15% during their reproductive years [6, 7]. Most ECs are detected at early stages when a curative resection is a feasible option in most cases. While 5-year survival rate in such patients approximates 95%, it decreases down to 16-45% in women with advanced late-stage EC [8–10].

EC can be classified into two types, type I and type II. Type I EC is endometrioid cancer (G1 or G2) with a hormonal etiology. The more aggressive type (type II EC) is a G3 malignancy with endometrioid, clear cell, or serous phenotype [11]. The type II tumors are estrogen-independent and associated with endometrial atrophy [12]. Most of type I ECs have a significantly better prognosis than the type II malignancies [13, 14]. Type I EC usually has a characteristic clinical profile; it is found in younger patients, most of whom have an identifiable source of excess estrogen, and endometrioid histologic subtypes are overwhelmingly more common [15]. The foremost risk factor for endometrial cancer, obesity, is strongly related to circulating estrogen levels in postmenopausal women and seems to be associated with an increased risk of EC of any type [16, 17]. An increase in the relative risk of EC in younger patients has been estimated at 1.59 per 5 kg/m^2^ BMI [18]. Other hormone-related risk factors for EC include nulliparity and older age at menopause [19, 20]. The list of presumable protective factors includes oral contraceptives, cigarette smoking, and older age at the last birth [20, 21]. Compared with women with type I EC, patients with type II EC are older, which more often have normal body weight and non-Caucasian heritage [16].

The majority of EC patients are eligible for primary surgical treatment: total hysterectomy or salpingo-oophorectomy, combined with lymphadenectomy if warranted by the presence of relevant risk factors [22]. The examination of surgical specimens provides information about cancer stage, prognosis, and the necessity of adjuvant treatment to decrease the likelihood of recurrence in patients with high-risk tumors [23]. EC is often spread via the lymphatics, with lymphatic metastases found in approximately 25% of patients with G3 tumors. Myometrial invasion up to two-thirds of the uterine wall thickness and vascular invasion are associated with the lymphatic spread in 22% and 15% of the cases, respectively. While only pelvic lymph nodes are involved in two-thirds of patients with lymphatic spread, more than 10% of women with EC may present with isolated positive para-aortal nodes [24]. Thus, hysterectomy with bilateral adnexectomy and pelvic and para-aortal lymphadenectomy is a recommended treatment option in patients with G3 tumors or/and myometrial invasion exceeding 50% of the uterine wall thickness [24–26]. In women with G1 or G2 tumors and more superficial myometrial invasion, hysterectomy with bilateral adnexectomy, but without lymphadenectomy, is a sufficient treatment option [27]. Radiotherapy is considered an effective adjuvant treatment option in EC [28].

Epithelial-mesenchymal transition (EMT) is a reversible cellular process during which epithelial cells depolarize, lose cell-cell contacts, and gain a spindle-mesenchymal morphology. EMT and an opposite process, mesenchymal to epithelial transition (MET), are essential for several developmental events in the embryo, such as gastrulation, formation of neural crest cells from the neural tube, mesoderm and heart valve formation, and palatogenesis [29]. EMT is associated with the loss of epithelial morphology and cytoskeletal reorganization, which allows the cells to gain migratory potential and higher invasiveness. Aside from being a physiological developmental mechanism, EMT also plays a role in tumor growth and progression to metastatic cancer. The EMT is triggered by several transcription factors in different signaling pathways. Several transcription factors, among them SNAIL, SLUG, TWIST, and ZEB, were shown to play essential roles in EMT control, contributing to downregulation of E-cadherin. The loss of E-cadherin expression is considered a major event in the EMT.

As compared with other malignancies, still little is known about the role of EMT transcription factors in EC [30]; the aim of this study was to analyze the expression of these factors: SNAIL, SLUG, TWIST1, TWIST2, ZEB1, and ZEB 2 in primary EC. Moreover, we verified whether the expression of the EMT transcription factors correlated with morphological and clinical characteristics of EC.

## 2. Methods

The study included 158 patients with EC, diagnosed, and treated at the Department of Obstetrics and Gynecology, Nicolaus Copernicus University, Collegium Medicum in Bydgoszcz (Poland). The mean age of the study patients was 66 years (range 37-87 years). The mean age at the last menstruation was 51 years (range 37-59 years), and the median parity amounted to 2 (range 0-10). The mean BMI for the study group was 29.56 kg/m^2^ (range 19-42 kg/m^2^). Clinicopathological characteristics of the study patients are summarized in Table 1. All patients underwent surgical treatment: total hysterectomy and lymphadenectomy. Adjuvant treatment consisted of radiotherapy, administered in line with the guidelines of Polish Oncological Society [31]. The relationship between the protein expression and survival was analyzed after minimum 5-year follow-up. Immunohistochemical studies were performed on archival formalin-fixed, paraffin-embedded (FFPE) tissue specimens, obtained from the Department of Clinical Pathomorphology Collegium Medicum, Nicolaus Copernicus University in Torun, Poland.

### 2.1. Tissue Macroarrays

Representative tumor areas were selected from the archival paraffin blocks (donor blocks). Tissue macroarrays (TMaAs) were obtained by transferring tissue fragments from the donor blocs to previously prepared recipient blocs. Each recipient block was composed of representative tissue from five EC patients. The representative tissue was reembedded in paraffin to obtain recipient blocks. Next, the TMaA block was cut into 4 *μ*m thick sections, using a rotary microtome (Accu-Cut® SRM™ 200; Sakura, Torrance, CA, USA). The sections were transferred on extra-adhesive slides (Superfrost Plus; Mensel-Glazer, Braunschweig, Germany) and left for one hour on a heating plate set at 60°C.

### 2.2. Immunohistochemical Staining (IHC)

Immunohistochemical staining was performed according to the protocol described previously [32], using the primary rabbit polyclonal anti-SNAIL (1 : 100, 45 min, NBP1-80022, Novus Biologicals) antibody, mouse monoclonal anti-SLUG (1 : 200, 30 min, NBP2-03886, Novus Biologicals) antibody, mouse monoclonal anti-TWIST1 (1 : 20, 60 min, ab50887, Abcam) antibody, mouse monoclonal anti-TWIST2 (1 : 200, 45 min, ab57997, Abcam) antibody, rabbit polyclonal anti-ZEB1 (1 : 500, 30 min, HPA027524, Sigma-Aldrich) antibody, and rabbit polyclonal anti-ZEB2 (1 : 100, 45 min, HPA003456, Sigma-Aldrich) antibody. The antibody complexes were detected using an EnVision Flex Anti-Mouse/Rabbit HRP-Labeled Polymer (Dako; Agilent Technologies, Inc.) and localized using 3,3′-diaminobenzidine (DAB) as the chromogen. The staining for SNAIL, SLUG, TWIST1, TWIST2, ZEB1, and ZEB2 was performed automatically in AutostainerLink48 (Dako; Agilent Technologies, Inc.). To standardize the immunohistochemical procedures, a series of positive and negative control reactions were carried out. Positive controls were tissue models in which the presence of analyzed antigens was indicated in reference sources (The Human Protein Atlas: https://www.proteinatlas.org.) as well as in the respective antibody datasheet. Negative control was obtained by substituting the primary antibody with a 1% bovine serum albumin (BSA) diluted in phosphate-buffered saline (PBS).

### 2.3. Evaluation of Immunohistochemical Reactions

The antibody-labeled slides were evaluated by two independent pathologists under a low-power (×20) ECLIPSE E800 light microscope (Nikon Instruments Europe, Amsterdam, Netherlands). In addition, low and negative-intensity slides were evaluated in high-power magnification (x40). The immunoexpression of analyzed proteins in EC macroarrays was quantified using Remmele-Stegner (IRS) scoring system. The IRS score for each macroarray spot was calculated by multiplying staining intensity (0 = negative, 1 = weakly positive, 2 = moderately positive, and 3 = strongly positive) by the proportion of positively stained cells (1 = 1–9%, 2 = 10–50%, 3 = 51–80%, and 4 = 81–100%); hence, the final scores might vary between 0 and 12. Statistical analysis included a mean IRS score for all tissue macroarrays [33].

### 2.4. Statistical Analysis

Statistical analysis was carried out with PQStat package, version 1.6.4.110. The effect of grouping clinicopathological variables on the immunoexpression of studied proteins was analyzed with the Mann-Whitney *U*-test. The relationship between analyzed proteins and established unfavorable prognostic factors in EC was estimated using univariate and multivariate logistic regression models. The relationship between the protein expression and survival was analyzed with univariate and multivariate Cox proportional-hazards models. Survivals of patients with weaker and stronger expressions of SLUG were compared with log-rank, Wilcoxon-Breslow-Gehan, and Taron-Ware tests. The results of all tests were considered significant at *p* < 0.05 and highly significant at *p* < 0.01.

### 2.5. Ethics

The protocol of the study was approved by the Local Bioethics Committee at the Nicolaus Copernicus University, Collegium Medicum in Bydgoszcz.

## 3. Results

**Figure 1:**
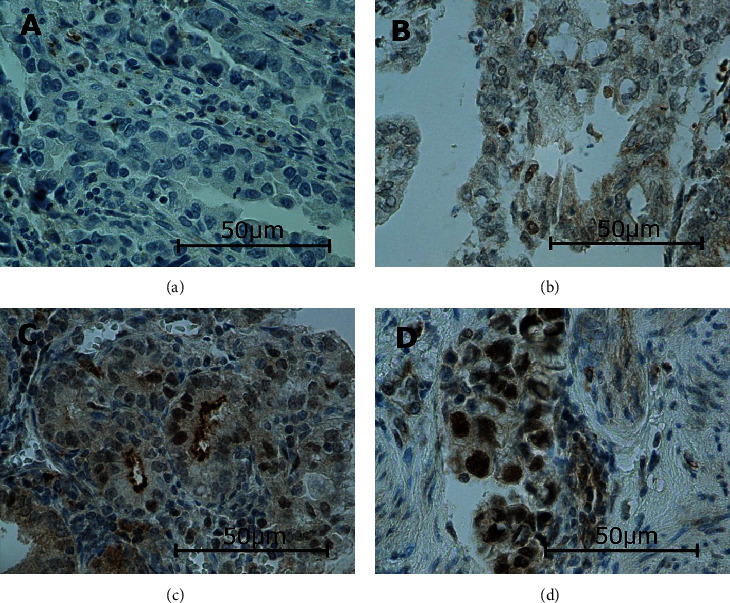
Levels of nuclear SNAIL expression in endometrial cancer: negative (a), weak (b), medium (c), and strong (d). Primary objective magnification: 40x.

**Figure 2:**
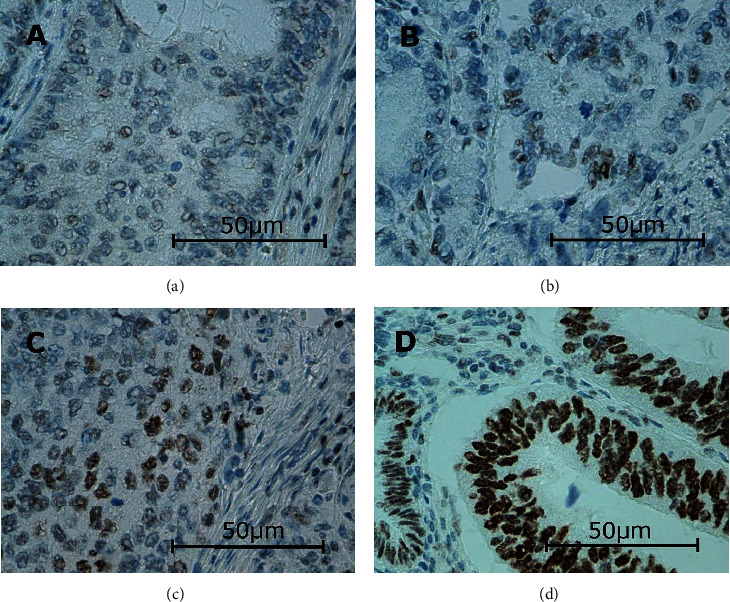
Levels of nuclear SLUG expression in endometrial cancer: negative (a), weak (b), medium (c), and strong (d). Primary objective magnification: 40x.

**Figure 3:**
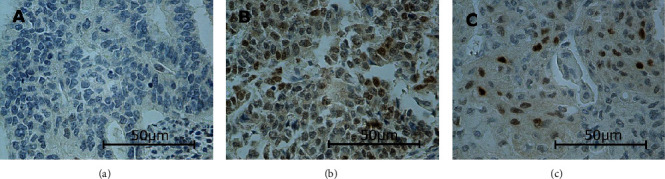
Levels of nuclear TWIST1 expression in endometrial cancer: negative (a), medium (b), and strong (c). Notice that although the conspicuous expression, it is always in few cells. There was no weak expression in our study. Primary objective magnification: 40x.

**Figure 4:**
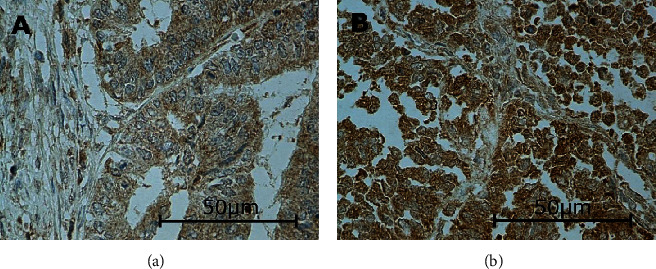
Levels of cytoplasmic TWIST2 expression in endometrial cancer: weak (a) and medium (b). There was neither negative nor strong expression in our study. Primary objective magnification: 40x.

**Figure 5:**
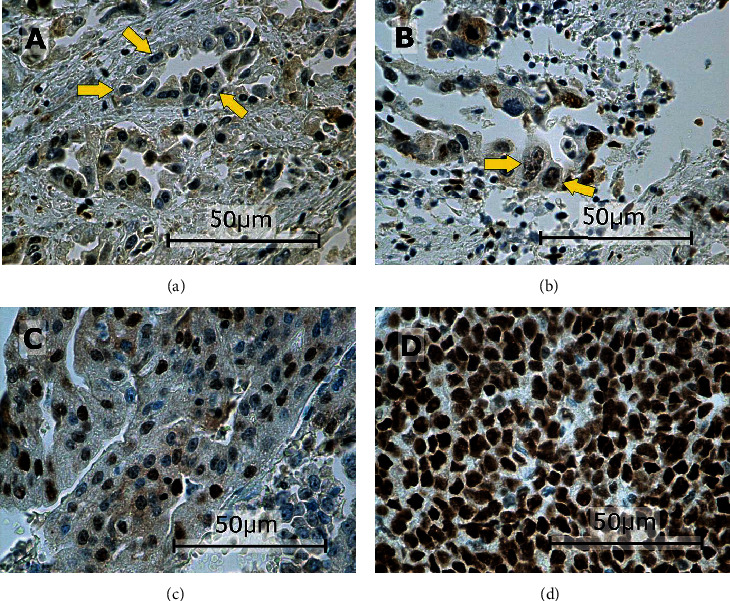
Levels of nuclear ZEB1 expression in endometrial cancer: negative ((a) arrows), weak ((b) arrows), medium (c), and strong (d). Primary objective magnification: 40x.

**Figure 6:**
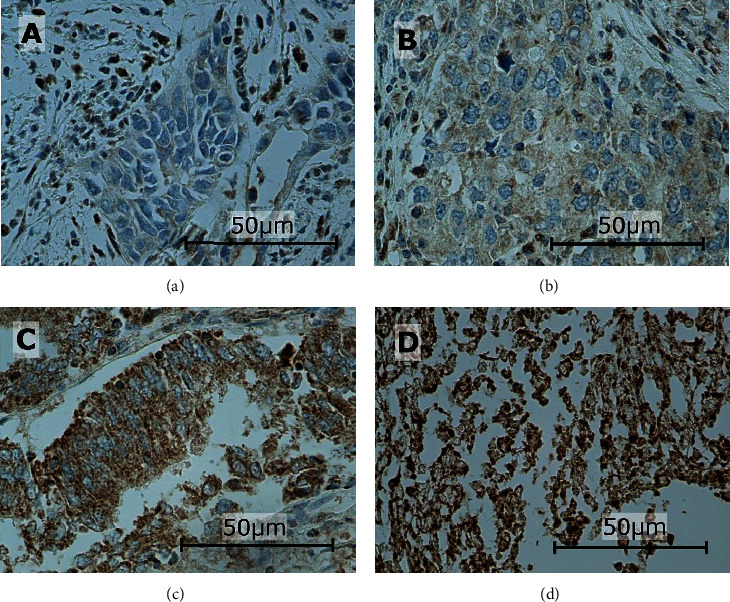
Levels of cytoplasmic ZEB2 expression in endometrial cancer: negative (a), weak (b), medium (c), and strong (d). Primary objective magnification: 40x.

In this study, the immunoexpressions of several EMT proteins: SLUG, SNAIL, TWIST1, TWIST2, ZEB1, and ZEB2, were evaluated in primary ECs. The percentages of EC specimens testing positively for the EMT transcription factors were 84.5% for SNAIL, 92.2% for SLUG, 10.9% for TWIST1, 100% for TWIST2, 89% for ZEB1, and 98% for ZEB2. In the case of SNAIL, SLUG, TWIST1, and ZEB1, the immunoexpression was found in the cell nuclei and in the case of TWIST2 and ZEB2 in the cytoplasm. Representative immunohistochemical reactions for various proteins are shown in Figures 1–6.

The expression of SLUG differed significantly (*p* < 0.05) depending on clinical FIGO stage. The expression in patients with FIGO stage III or IV was significantly higher than that in those with less-advanced ECs. The SLUG expression was also significantly higher (*p* < 0.01) in type II ECs than in type I malignancies. No statistically significant differences in the SLUG expression were found after stratifying the immunohistochemical results according to histological grade, lymphovascular space invasion (LVSI), cervical invasion, and lymph node involvement. The expressions of SLUG in patients with myometrial invasion ≥ 50% of the uterine wall thickness and adnexal involvement were significantly higher (*p* < 0.05) than those in those without these unfavorable prognostic factors. The expression of SLUG was also significantly higher in patients with distant metastases (*p* < 0.05) (Table 2). No significant associations were found between the clinicopathological characteristics of EC patients and the expressions of TWIST1, TWIST2, ZEB1, and SNAIL.

ZEB2 expression was significantly higher in patients with adnexal involvement than in those without. Other clinicopathological variables (FIGO stage, Bokhman type, histological grade, LVSI, myometrial invasion ≥ 50% of the uterine wall thickness, cervical invasion, lymph node involvement, and distant metastases) did not exert a significant effect on ZEB2 expression (Table 3).

SLUG and ZEB2 expressions were identified as significant predictors of higher FIGO stages (III or IV) on univariate analysis (*p* < 0.05). Patients with FIGO stage III or IV were more likely to present with SLUG and ZEB2 overexpression. Multivariate analysis identified SLUG overexpression as the only significant independent predictor of the higher clinical stage (*p* < 0.05) (Tables 4 and 5).

The overexpression of SLUG was also a significant predictor of more aggressive type II EC on both univariate and multivariate analyses (*p* < 0.01). Another independent predictor identified on the multivariate analysis was ZEB2 overexpression which turned out to be associated with a lesser likelihood of type II EC (*p* < 0.05) (Tables 6 and 7).

Univariate analysis demonstrated that the overexpression of SLUG was associated with a significantly higher likelihood of myometrial invasion ≥ 50% of the uterine wall thickness (*p* < 0.05). Multivariate analysis confirmed that the overexpression of SLUG was a significant independent predictor of the deep myometrial invasion (*p* < 0.01); the lesser likelihood of the deep myometrial invasion was in turn independently predicted by TWIST2 overexpression (*p* < 0.05) (Tables 8 and 9). The overexpression of SLUG and ZEB2 was shown to be significant predictors of adnexal involvement on univariate analysis (*p* < 0.05) (Tables 10 and 11). The overexpression of SLUG was also identified as the only significant predictor of distant metastases on both univariate and multivariate analyses (*p* < 0.05) (Tables 12 and 13). None of the analyzed proteins turned out to be a significant (*p* > 0.05) predictor of LVSI, cervical invasion, and lymph node involvement.

**Figure 7:**
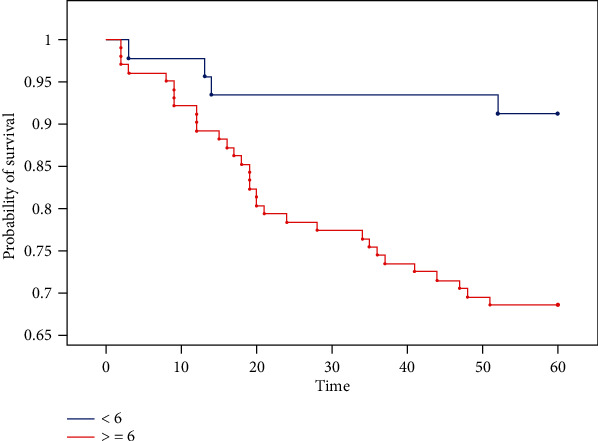
Kaplan-Meier curves for overall survival in patients with endometrial cancer stratified by SLUG expression. The Kaplan-Meier plots illustrate statistically significant differences (*p* < 0.05) in the probability of survival in patients with SLUG expression ≥ 6 and <6 (time/months). Logrank chi^2^ = 8, 2801,df = 1, and*p* = 0.0040. Wilcoxon-Breslow-Gehanchi^2^ = 8, 0659,df = 1, and*p* = 0.0045. Taron-Warechi^2^ = 8, 1876,df = 1, and*p* = 0, 0042.

Both univariate and multivariate analyses demonstrated that SLUG expression was the only predictor of 5-year survival in the study group. The overexpression of SLUG was associated with a significant increase in mortality hazard on univariate analysis (*p* < 0.05) and was shown to be a highly significant predictor of death on multivariate analysis (*p* < 0.01) (Tables 14 and 15). The probability of survival in patients with SLUG expressions < 6 and ≥6 is depicted in Figure 7. Highly significant differences in the survivals of the two groups were confirmed with log-rank, Wilcoxon-Breslow-Gehan, and Taron-Ware tests (*p* < 0.01).

## 4. Discussion

Tumor microenvironment parameters, in particular, changes in tumor cells-stromal cells interactions, play a crucial role in the development of epithelial malignancies [34]. Mesenchymal cells are essential for the control of epithelial growth, differentiation and function, and abnormal mesenchymal-epithelial interactions found in various human tumors, including EC [35, 36]. Metastatic spread results from a series of linked, sequential, and selective steps involving cell migration, invasion, adhesion, and proliferation, as well as angiogenesis. Invasion, which is a critical step in the metastatic cascade, requires the interaction of tumor cells with their environment [37]. EMT is the essential initial step of malignant transformation, during which the cells acquire invasiveness and metastatic potential by losing epithelial polarity and reducing intercellular adhesion [38, 39]. Many studies showed that EMT is involved in tumorigenesis and development of EC [40]. Downregulation of E-cadherin is considered a classical molecular switch for EMT and has been implicated in the invasion and spread of EC [41, 42].

Several transcription factors, among them the members of SLUG, SNAIL, ZEB, and TWIST families, have been implicated in the transcriptional repression of E-cadherin in a broad spectrum of human cancers and were shown to be associated with aggressive tumor behavior and poor prognosis [43–45]. In our present study, the expression of the EMT transcription factors was found in the vast majority of tissue samples, which implies that EMT plays a critical role in endometrial carcinogenesis. Moreover, the expression of SLUG turned out to be significantly higher in patients with FIGO stages III and IV, as well as in those with type II EC. It needs to be emphasized that we determined the expressions of EMT transcription factors in a relatively large group of patients with advanced EC. EMT, promoted by several transcription factors, causes downregulation of epithelial marker genes and contributes to the establishment of a mesenchymal phenotype [46]. SNAIL and SLUG, belonging to the group of zinc finger-type proteins, are regarded as major EMT inducers that inhibit the transcription of cell adhesion molecules, among them is E-cadherin [47]. As potent E-cadherin repressors, those proteins contribute to the loss of tight junctions between epithelial cells and initiate EMT, which facilitates cancer cell invasion and formation of distant metastases [48, 49]. The EMT signaling pathways may be activated by several cytokines or growth factors present in the local microenvironment, followed by the interaction with transforming growth factor-beta (TGF-*β*), bone morphogenetic protein, Wnt/*β*-catenin pathway, Notch, Hedgehog, and RTKs [47, 50]. Some signaling pathways, such as the phosphoinositide 3-kinase/protein kinase B (PI3K/AKT) pathway, upregulate SNAIL and SLUG, whereas the estrogen receptor is known to repress the transcription of those proteins [51, 52]. Importantly, the overexpression of SLUG can be mediated by various mechanisms; for example, 17b-estradiol is known to increase the expression of vimentin and SLUG and to decrease the expression of E-cadherin, which leads to the inhibition of EMT and estrogen-induced EMT in EC cells [53]. Also, transforming growth factor-beta (TGF-*β*) was shown to be an EMT inducer which downregulates E-cadherin, upregulates SLUG, and stimulates cell invasion in EC; the TGF-b-induced cell invasion could be prevented through SLUG depletion caused by siRNA knockdown [54].

Our present study demonstrated that myometrial invasion exceeding 50% of the uterine wall thickness and adnexal involvement were associated with a significant increase in SLUG expression. In turn, the overexpression of TWIST2 correlated with a lesser risk of deep myometrial infiltration in EC patients. A significant increase in SLUG expression was also observed in patients with distant metastases. Adnexal involvement was also shown to be associated with a significant increase in ZEB2 expression. Our findings are consistent with the results published by other authors, according to whom the deep myometrial invasion was associated with SLUG overexpression and a concomitant decrease in E-cadherin level [55]. Recent studies showed that SLUG plays a role not only in cancer spread but also in cancer stemness [56, 57]; this suggests that the protein might be involved in the early stages of cancer progression. SLUG is known to suppress both p53-dependent and p53-independent apoptotic pathways [58]. Tumors expressing SLUG might display some characteristics of cancer stem cells, such as therapeutic resistance and ability to recur. The overexpression of E-cadherin transcription repressors, TWIST, SNAIL, and SLUG, was observed in both EC cell lines and tumor samples, and downregulation of E-cadherin was demonstrated in either endometrioid or nonendometrioid ECs. Hence, SLUG might play a significant role in the development of therapeutic resistance and contribute to poor prognosis in a subset of high-grade ECs. Despite complete resection, patients with the extrauterine disease are assumed to present with micrometastases, and thus, are at increased risk of tumor recurrence; if the primary malignancy overexpresses SLUG, the recurrent tumor is likely to be resistant to adjuvant therapy [59]. ZEB1 and ZEB2 are transcriptional repressors of E-cadherin in a miR-200-dependent mechanism. The inhibition of miR-200 results in the downregulation of E-cadherin through upregulation of its transcriptional repressors, ZEB1 and ZEB2. Hence, the ZEB proteins and miR-200 are considered a driving force for cancer progression and spread by controlling the state of cancer stem cells [60, 61].

In our present study, SLUG was the only protein, the expression of which was identified as a significant predictor of 5-year survival on both univariate and multivariate analyses. The overexpression of SLUG was associated with a significant increase in mortality hazard. This implies that the expression of SLUG may be an important determinant of survival in EC. Some clinicopathological parameters, identified as independent predictors of overall survival and disease-free survival in EC, among them patient age, tumor grade, histological type, and LVSI, are considered decisive factors during the selection of surgical treatment and adjuvant therapy options [62]. Established prognostic factors for the recurrence and spread of EC include surgical FIGO stage, tumor grade, histological type, and myometrial and lymphovascular invasion [63, 64]. Our findings suggest that the inclusion of SLUG and ZEB2 expressions in the armamentarium of routinely performed immunohistochemical tests might contribute to more accurate prognosis and facilitate the planning of adjuvant therapy, especially in patients with advanced clinical stages of type I EC and those with type II malignancies.

## 5. Conclusions

1. Selected proteins of the EMT pathway play a role in endometrial carcinogenesis
2. SLUG and ZEB2 expressions in the primary tumor might predict clinical outcomes in EC and drive therapeutic decisions regarding adjuvant treatment in patients with this malignancy

## Figures and Tables

**Table 1:** Clinical characteristics of study subjects.

Feature		*N*	%
Stage	I	75	47
	II	34	22
	III	38	24
	IV	11	7

Grade	1	9	6
	2	105	72
	3	32	22

Bokham subtype	I	118	75
	II	40	25

LVSI	Negative	129	82
	Positive	29	18

Meta at lymph nodes	No	124	82
	Yes	28	18

Distant metastases	No	137	91
	Yes	14	9

Infiltration of myometrium	<50%	48	30
	≧50%	110	70

Infiltration of cervix	No	95	61
	Yes	62	39

Infiltration of adnexa	No	134	87
	Yes	20	13

LVSI: lymphovascular space invasion.

**Table 2:** SLUG expression according to clinicopathological features.

		Mean	Standard deviation (SD)	Minimum	Lower quartile (Q1)	Median (Me)	Upper quartile (Q3)	Maximum	Mann-Whitney *U*-test
Total		7.30	3.93	0	4	8	12	12	—

FIGO stage	I+II	6.82	3.85	0	4	7	9	12	*Z* = 2.3333 *p* = 0.0196
	III+IV	8.35	3.94	0	6	8.5	12	12	

Bokhman subtype	I	6.64	3.85	0	4	6	9	12	*Z* = 3.7265 *p* = 0.0002
	II	9.18	3.57	0	8	10.5	12	12	

Grading	G1+G2	7.08	3.98	0	4	8	12	12	*Z* = 0.6527 *p* = 0.5140
	G3	7.68	3.97	0	4	8	12	12	

LVSI	Positive	7.15	3.94	0	4	8	12	12	*Z* = 0.9659 *p* = 0.3341
	Negative	7.96	3.91	0	6	8	12	12	

Myometrial invasion	<50%	6.04	3.92	0	3.25	6	9	12	*Z* = 2.4943 *p* = 0.0126
	≥50%	7.83	3.83	0	4	8	12	12	

Cervical involvement	No	6.86	4.00	0	4	8	12	12	*Z* = 1.6616 *p* = 0.0966
	Yes	8.00	3.78	0	4	8	12	12	

Infiltration of adnexa	No	7.05	3.91	0	4	8	12	12	*Z* = 2.135 *p* = 0.0328
	Yes	9.00	3.39	0	7.5	9	12	12	

Lymph nodes metastases	No	6.94	3.81	0	4	8	9	12	*Z* = 1.757 *p* = 0.0789
	Yes	8.33	4.43	0	5	12	12	12	

Distant metastases	No	6.99	3.99	0	4	8	12	12	*Z* = 2.5126 *p* = 0.012
	Yes	9.86	2.66	6	8	12	12	12	

FIGO: Federation Internationale de Gynecologie et d'Obstetrique; LVSI: lymphovascular space invasion.

**Table 3:** ZEB 2 expression according to clinicopathological features.

		Mean	Standard deviation (SD)	Minimum	Lower quartile (Q1)	Median (me)	Upper quartile (Q3)	Maximum	Mann-Whitney *U*-test
Total		5.13	2.02	0	4	4	8	8	—

FIGO stage	I+II	4.91	2.02	0	4	4	7	8	*Z* = 1.9126 *p* = 0.0558
	III+IV	5.63	1.95	3	4	4	8	8	

Bokhman subtype	I	5.04	1.97	0	4	4	8	8	*Z* = 1.2604 *p* = 0.2075
	II	5.65	2.11	2	4	4	8	8	

Grading	G1+G2	5.25	2.09	0	4	4	8	8	*Z* = 1.4442 *p* = 0.1487
	G3	4.78	1.78	1	4	4	4.5	8	

LVSI	Positive	5.20	2.09	0	4	4	8	8	*Z* = 0.9575 *p* = 0.3383
	Negative	4.79	1.66	2	4	4	4.5	8	

Myometrial invasion	<50%	5.08	2.01	0	4	4	8	8	*Z* = 0.5308 *p* = 0.5955
	≥50%	5.41	1.97	3	4	4	8	8	

Cervical involvement	No	5.11	2.00	0	4	4	8	8	*Z* = 0.5294 *p* = 0.5965
	Yes	5.50	2.03	3	4	4	8	8	

Infiltration of adnexa	No	5.22	2.01	1	4	4	8	8	*Z* = 0.2918 *p* = 0.7704
	Yes	5.09	2.03	0	4	4	8	8	

Lymph nodes metastases	No	4.92	1.90	0	4	4	6	8	*Z* = 1.7265 *p* = 0.0843
	Yes	5.46	2.17	0	4	4	8	8	

Distant metastases	No	5.01	2.01	0	4	4	8	8	*Z* = 2.1077 *p* = 0.0351
	Yes	6.00	1.95	4	4	6	8	8	

FIGO: Federation Internationale de Gynecologie et d'Obstetrique; LVSI: lymphovascular space invasion.

**Table 4:** Results of univariate logistic regression analyses examining the effects of selected proteins on the incidence of advanced FIGO stage (FIGO III+IV).

	*b* coefficient	*p* value	Odds ratio	-95% CI	+95% CI
Intercept	-1,144	0,0527	0,3186	0,1001	1,0135
TWIST 2	0,0532	0,5414	1,0546	0,8891	1,251
Intercept	-0,7336	<0,0001	0,4802	0,3376	0,683
TWIST 1	-0,2509	0,2406	0,7781	0,5118	1,1831
Intercept	-1,588	0,0001	0,2043	0,091	0,4588
SLUG	0,1046	0,0264	1,1103	1,0123	1,2177
Intercept	-1,7366	0,0005	0,1761	0,0659	0,471
ZEB 2	0,1777	0,0417	1,1945	1,0067	1,4173
Intercept	-0,5327	0,138	0,587	0,2904	1,1868
ZEB 1	-0,0623	0,3993	0,9396	0,813	1,086
Intercept	-0,5927	0,0709	0,5528	0,2906	1,0518
SNAIL	-0,0548	0,4799	0,9467	0,8131	1,1022

CI: confidence interval.

**Table 5:** Results of multivariate logistic regression analyses examining the effects of selected proteins on the incidence of advanced FIGO stage (FIGO III+IV).

	*b* coefficient	*p* value	Odds ratio	-95% CI	+95% CI
Intercept	-2,0322	0,0111	0,131	0,0273	0,6285
TWIST 2	0,0412	0,6722	1,042	0,8611	1,2611
TWIST 1	-0,2112	0,3781	0,8096	0,5062	1,2949
SLUG	0,1036	0,0377	1,1092	1,0059	1,223
ZEB 2	0,1375	0,1548	1,1474	0,9494	1,3866
ZEB 1	-0,0462	0,5792	0,9549	0,8111	1,1241
SNAIL	-0,0752	0,4056	0,9275	0,7768	1,1075

CI: confidence interval.

**Table 6:** Results of univariate logistic regression analyses examining the effects of selected proteins on the incidence of subtype 2 by Bokhman.

	*b* coefficient	*p* value	Odds ratio	-95% CI	+95% CI
Intercept	-0,657	0,267	0,5184	0,1625	1,6539
TWIST 2	-0,063	0,4825	0,9389	0,7875	1,1195
Intercept	-1,0878	<0,0001	0,337	0,2313	0,491
TWIST 1	0,0843	0,5438	1,0879	0,8287	1,4282
Intercept	-2,5356	<0,0001	0,0792	0,029	0,2161
SLUG	0,1866	0,0007	1,2052	1,0818	1,3427
Intercept	-0,4486	0,3671	0,6385	0,2409	1,6926
ZEB 2	-0,1212	0,1991	0,8858	0,7362	1,0659
Intercept	-0,8881	0,0193	0,4115	0,1955	0,8659
ZEB 1	-0,0388	0,6175	0,962	0,8262	1,12
Intercept	-1,2654	0,0004	0,2821	0,141	0,5647
SNAIL	0,0579	0,4627	1,0596	0,9079	1,2366

CI: confidence interval.

**Table 7:** Results of multivariate logistic regression analyses examining the effects of selected proteins on the incidence of subtype 2 by Bokhman.

	*b* coefficient	*p* value	Odds ratio	-95% CI	+95% CI
Intercept	-1,3111	0,1273	0,2695	0,05	1,4538
TWIST 2	-0,0371	0,7225	0,9635	0,785	1,1827
TWIST 1	0,1521	0,3927	1,1643	0,8215	1,6502
SLUG	0,2277	0,0001	1,2557	1,1192	1,4089
ZEB 2	-0,2412	0,0307	0,7857	0,6313	0,9778
ZEB 1	-0,1183	0,1987	0,8884	0,7417	1,0641
SNAIL	0,0999	0,2631	1,1051	0,9277	1,3165

CI: confidence interval.

**Table 8:** Results of univariate logistic regression analyses examining the effects of selected proteins on the incidence of myometrial invasion > =50%.

	*b* coefficient	*p* value	Odds ratio	-95% CI	+95% CI
Intercept	1,7835	0,0048	5,9505	1,7245	20,5328
TWIST 2	-0,1413	0,1219	0,8682	0,7258	1,0385
Intercept	0,877	<0,0001	2,4036	1,6783	3,4424
TWIST 1	-0,0397	0,7758	0,9611	0,7315	1,2628
Intercept	0,0345	0,9225	1,0352	0,5161	2,0761
SLUG	0,1178	0,0108	1,1251	1,0276	1,2318
Intercept	1,0222	0,0349	2,7794	1,0753	7,184
ZEB 2	-0,0309	0,7225	0,9695	0,8173	1,1501
Intercept	0,3432	0,3423	1,4095	0,6941	2,8622
ZEB 1	0,1223	0,1104	1,1301	0,9725	1,3132
Intercept	0,9194	0,006	2,5079	1,3015	4,8324
SNAIL	-0,0178	0,8159	0,9824	0,846	1,1408

CI: confidence interval.

**Table 9:** Results of multivariate logistic regression analyses examining the effects of selected proteins on the incidence of myometrial invasion > =50%.

	*b* coefficient	*p* value	Odds ratio	-95% CI	+95% CI
Intercept	0,8942	0,2557	2,4455	0,5232	11,4298
TWIST 2	-0,2234	0,0345	0,7998	0,6502	0,9838
TWIST 1	-0,123	0,4994	0,8843	0,6189	1,2635
SLUG	0,133	0,0085	1,1423	1,0345	1,2614
ZEB 2	-0,0185	0,8488	0,9817	0,8117	1,1873
ZEB 1	0,1679	0,0548	1,1829	0,9966	1,4039
SNAIL	-0,0276	0,7500	0,9728	0,8211	1,1526

CI: confidence interval.

**Table 10:** Results of univariate logistic regression analyses examining the effects of selected proteins on the incidence of infiltration of adnexa.

	*b* coefficient	*p* value	Odds ratio	-95% CI	+95% CI
Intercept	-2,1811	0,0098	0,1129	0,0216	0,5912
TWIST 2	0,0463	0,7067	1,0474	0,8229	1,3333
Intercept	-1,8675	<0,0001	0,1545	0,0949	0,2515
TWIST 1	-0,0368	0,862	0,9639	0,6365	1,4597
Intercept	-3,0314	<0,0001	0,0482	0,0131	0,1771
SLUG	0,1438	0,0408	1,1546	1,006	1,3252
Intercept	-3,2094	<0,0001	0,0404	0,0094	0,1733
ZEB 2	0,2418	0,0437	1,2736	1,0068	1,6109
Intercept	-1,3751	0,0033	0,2528	0,1011	0,632
ZEB 1	-0,1218	0,2366	0,8853	0,7237	1,0831
Intercept	-1,9968	<0,0001	0,1358	0,0527	0,3496
SNAIL	0,0332	0,7617	1,0337	0,8343	1,2807

CI: confidence interval.

**Table 11:** Results of multivariate logistic regression analyses examining the effects of selected proteins on the incidence of infiltration of adnexa.

	*b* coefficient	*p* value	Odds ratio	-95% CI	+95% CI
Intercept	-3,4897	0,0026	0,0305	0,0031	0,2961
TWIST 2	-0,0102	0,9412	0,9899	0,7554	1,2972
TWIST 1	0,028	0,9102	1,0284	0,6319	1,6738
SLUG	0,1343	0,0619	1,1438	0,9934	1,317
ZEB 2	0,1805	0,1718	1,1978	0,9246	1,5517
ZEB 1	-0,1547	0,2146	0,8567	0,671	1,0937
SNAIL	0,0667	0,6059	1,069	0,8298	1,3771

CI: confidence interval.

**Table 12:** Results of univariate logistic regression analyses examining the effects of selected proteins on the incidence of distant metastases.

	*b* coefficient	*p* value	Odds ratio	-95% CI	+95% CI
Intercept	-2,8899	0,0045	0,0556	0,0076	0,4076
TWIST 2	0,0967	0,5085	1,1015	0,8271	1,4669
Intercept	-2,1927	<0,0001	0,1116	0,0634	0,1964
TWIST 1	-0,277	0,5042	0,7581	0,3363	1,7088
Intercept	-4,1797	<0,0001	0,0153	0,0024	0,0972
SLUG	0,2256	0,0153	1,253	1,0441	1,5037
Intercept	-2,767	0,0006	0,0628	0,0129	0,3063
ZEB 2	0,0958	0,4898	1,1006	0,8385	1,4444
Intercept	-2,926	<0,0001	0,0536	0,0148	0,1944
ZEB 1	0,1408	0,2304	1,1512	0,9146	1,449
Intercept	-2,2616	<0,0001	0,1042	0,0369	0,2939
SNAIL	0,0028	0,9816	1,0028	0,7915	1,2705

CI: confidence interval.

**Table 13:** Results of multivariate logistic regression analyses examining the effects of selected proteins on the incidence of distant metastases.

	*b* coefficient	*p* value	Odds ratio	-95% CI	+95% CI
Intercept	-5,1986	0,0012	0,0055	0,0002	0,1295
TWIST 2	0,0514	0,7505	1,0528	0,7668	1,4455
TWIST 1	-0,3348	0,4795	0,7155	0,2828	1,8098
SLUG	0,2357	0,0171	1,2658	1,0428	1,5363
ZEB 2	0,0326	0,8438	1,0332	0,7467	1,4295
ZEB 1	0,1489	0,2665	1,1606	0,8925	1,5093
SNAIL	-0,056	0,6888	0,9455	0,7187	1,2438

CI: confidence interval.

**Table 14:** Prognostic factors for overall survival selected by Cox's univariate analysis.

	*b* coefficient	*p* value	HR	-95% CI	+95% CI
TWIST 2	0,0623	0,4678	1,0643	0,8995	1,2592
TWIST 1	-14,149	0,9875	0,0000	0,0000	0,0000
SLUG	0,1157	0,0138	1,1226	1,0239	1,2309
ZEB 2	-0,0274	0,7393	0,973	0,828	1,1434
ZEB 1	0,0558	0,4176	1,0574	0,9239	1,2102
SNAIL	0,0363	0,6143	1,037	0,9004	1,1943

CI: confidence interval; HR: hazard ratio.

**Table 15:** Prognostic factors for overall survival selected by Cox's multivariate analysis.

	*b* coefficient	*p* value	HR	-95% CI	+95% CI
TWIST 2	0,0479	0,601	1,049	0,8767	1,2552
TWIST 1	-13,63	0,9816	1,2E-6	0	NA
SLUG	0,1374	0,0052	1,1472	1,0418	1,2633
ZEB 2	-0,1108	0,2217	0,8951	0,7494	1,0692
ZEB 1	0,0126	0,862	1,0127	0,8783	1,1677
SNAIL	0,0648	0,4437	1,0669	0,904	1,2592

CI: confidence interval; HR: hazard ratio.

## Data Availability

The data used to support the findings of this study are available from the corresponding author upon request.

## References

[1] Ferlay J., Soerjomataram I., Dikshit R. (2015). Cancer incidence and mortality worldwide: sources, methods and major patterns in GLOBOCAN 2012. *International Journal of Cancer*.

[2] Siegel R. L., Miller K. D., Jemal A. (2017). Cancer statistics, 2017. *CA: a Cancer Journal for Clinicians*.

[3] Lortet-Tieulent J., Ferlay J., Bray F., Jemal A. (2018). International patterns and trends in endometrial cancer incidence, 1978-2013. *Journal of the National Cancer Institute*.

[4] Smittenaar C. R., Petersen K. A., Stewart K., Moitt N. (2016). Cancer incidence and mortality projections in the UK until 2035. *British Journal of Cancer*.

[5] Sheikh M. A., Althouse A. D., Freese K. E. (2014). USA endometrial cancer projections to 2030: should we be concerned?. *Future Oncology*.

[6] Siegel R. L., Devesa S. S., Cokkinides V., Ma J., Jemal A. (2013). State-level uterine corpus cancer incidence rates corrected for hysterectomy prevalence, 2004 to 2008. *Cancer Epidemiology, Biomarkers & Prevention*.

[7] DeSantis C. E., Lin C. C., Mariotto A. B. (2014). Cancer treatment and survivorship statistics, 2014. *CA: A Cancer Journal for Clinicians*.

[8] Yeramian A., Moreno-Bueno G., Dolcet X. (2013). Endometrial carcinoma: molecular alterations involved in tumor development and progression. *Oncogene*.

[9] Bray F., Ferlay J., Soerjomataram I., Siegel R. L., Torre L. A., Jemal A. (2018). Global cancer statistics 2018: GLOBOCAN estimates of incidence and mortality worldwide for 36 cancers in 185 countries. *CA: A Cancer Journal for Clinicians*.

[10] Weiderpass E., Antoine J., Bray F. I., Oh J. K., Arbyn M. (2014). Trends in corpus uteri cancer mortality in member states of the European Union. *European Journal of Cancer*.

[11] Brinton L. A., Felix A. S., McMeekin D. S. (2013). Etiologic heterogeneity in endometrial cancer: evidence from a Gynecologic Oncology Group trial. *Gynecologic Oncology*.

[12] Bokhman J. V. (1983). Two pathogenetic types of endometrial carcinoma. *Gynecologic Oncology*.

[13] Alkushi A., Abdul-Rahman Z. H., Lim P. (2005). Description of a novel system for grading of endometrial carcinoma and comparison with existing grading systems. *The American Journal of Surgical Pathology*.

[14] Scholten A. N., Smit V. T., Beerman H., van Putten W. L., Creutzberg C. L. (2004). Prognostic significance and interobserver variability of histologic grading systems for endometrial carcinoma. *Cancer*.

[15] Soliman P. T., Oh J. C., Schmeler K. M. (2005). Risk factors for young premenopausal women with endometrial cancer. *Obstetrics and Gynecology*.

[16] Setiawan V. W., Yang H. P., Pike M. C. (2013). Type I and II endometrial cancers: have they different risk factors?. *Journal of Clinical Oncology*.

[17] Madigan M. P., Troisi R., Potischman N., Dorgan J. F., Brinton L. A., Hoover R. N. (1998). Serum hormone levels in relation to reproductive and lifestyle factors in postmenopausal women (United States). *Cancer Causes & Control*.

[18] Topuz S., Sozen H., Vatansever D. (2016). Do obesity and age effect the clinicopathological features and survival outcomes in premenopausal women with endometrial cancer?. *European Journal of Gynaecological Oncology*.

[19] Schonfeld S. J., Hartge P., Pfeiffer R. M. (2013). An aggregated analysis of hormonal factors and endometrial cancer risk by parity. *Cancer*.

[20] Yang H. P., Wentzensen N., Trabert B. (2013). Endometrial cancer risk factors by 2 main histologic subtypes. *American Journal of Epidemiology*.

[21] Setiawan V. W., Pike M. C., Karageorgi S. (2012). Age at last birth in relation to risk of endometrial cancer: pooled analysis in the epidemiology of endometrial cancer consortium. *American Journal of Epidemiology*.

[22] Uterine neoplasms *NCCN Clinical Practice Guidelines in Oncology*.

[23] Wright J. D., Medel N. I. B., Sehouli J., Fujiwara K., Herzog T. J. (2012). Contemporary management of endometrial cancer. *Lancet*.

[24] Mariani A., Dowdy S. C., Cliby W. A. (2008). Prospective assessment of lymphatic dissemination in endometrial cancer: a paradigm shift in surgical staging. *Gynecologic Oncology*.

[25] Gao H., Zhang Z. (2015). Laparoscopy versus laparotomy in the treatment of high-risk endometrial cancer. *Medicine*.

[26] Mariani A., Webb M. J., Galli L., Podratz K. C. (2000). Potential therapeutic role of para-aortic lymphadenectomy in node-positive endometrial cancer. *Gynecologic Oncology*.

[27] Eltabbakh G. H., Piver M. S., Hempling R. E., Shin K. H. (1997). Excellent long-term survival and absence of vaginal recurrences in 332 patients with low-risk stage I endometrial adenocarcinoma treated with hysterectomy and vaginal brachytherapy without formal staging lymph node sampling: report of a prospective trial. *International Journal of Radiation Oncology • Biology • Physics*.

[28] Mariani A., Webb M. J., Keeney G. L., Haddock M. G., Calori G., Podratz K. C. (2000). Low-risk corpus cancer: is lymphadenectomy or radiotherapy necessary?. *American Journal of Obstetrics and Gynecology*.

[29] Larue L., Bellacosa A. (2005). Epithelial-mesenchymal transition in development and cancer: role of phosphatidylinositol 3′ kinase/AKT pathways. *Oncogene*.

[30] Colas E., Pedrola N., Devis L. (2012). The EMT signaling pathways in endometrial carcinoma. *Clinical and Translational Oncology*.

[31] Sznurkowski J. J., Knapp P., Bodnar L. (2017). Recommendations of the Polish Gynecological Oncology Society for the diagnosis andtreatment of endometrial cancer. *Current Gynecologic Oncology*.

[32] Sadlecki P., Jóźwicki J., Antosik P., Grabiec M. (2018). Expression of selected epithelial-mesenchymal transition transcription factors in serous borderline ovarian tumors and type I ovarian cancers. *Tumor Biology*.

[33] Remmele W., Stegner H. E. (1987). Recommendation for uniform definition of an immunoreactive score (IRS) for immunohistochemical estrogen receptor detection (ER-ICA) in breast cancer tissue. *Pathologe*.

[34] Shi M., Zhang H., Li M. (2011). Normal endometrial stromal cells regulate survival and apoptosis signaling through PI3K/AKt/ Survivin pathway in endometrial adenocarcinoma cells in vitro. *Gynecologic Oncology*.

[35] Sadlecki P., Grabiec M., Grzanka D., Jóźwicki J., Antosik P., Walentowicz-Sadlecka M. (2019). Expression of zinc finger transcription factors (ZNF143 and ZNF281) in serous borderline ovarian tumors and low-grade ovarian cancers. *J Ovarian Res*.

[36] Arnold Julia T., Lessey Bruce A., Seppala M., Kaufman D. G. (2002). Effect of normal endometrial stroma on growth and differentiation in Ishikawa endometrial adenocarcinoma cells. *Cancer Research*.

[37] Woodhouse E. C., Chuaqui R. F., Liotta L. A. (1997). General mechanisms of metastasis. *Cancer*.

[38] Tania M., Khan M. A., Fu J. (2014). Epithelial to mesenchymal transition inducing transcription factors and metastatic cancer. *Tumor Biology*.

[39] Christiansen J. J., Rajasekaran A. K. (2006). Reassessing epithelial to mesenchymal transition as a prerequisite for carcinoma invasion and metastasis. *Cancer Research*.

[40] Singh M., Spoelstra N. S., Jean A. (2008). ZEB1 expression in type I vs type II endometrial cancers: a marker of aggressive disease. *Modern Pathology*.

[41] Matsuzaki S., Darcha C. (2012). Epithelial to mesenchymal transition-like and mesenchymal to epithelial transition-like processes might be involved in the pathogenesis of pelvic endometriosis. *Human Reproduction*.

[42] Cano A., Pérez-Moreno M. A., Rodrigo I. (2000). The transcription factor Snail controls epithelial-mesenchymal transitions by repressing E-cadherin expression. *Nature Cell Biology*.

[43] Peinado H., Olmeda D., Cano A. (2007). Snail, Zeb and bHLH factors in tumour progression: an alliance against the epithelial phenotype?. *Nature Reviews. Cancer*.

[44] de Herreros A. G., Peiró S., Nassour M., Savagner P. (2010). Snail family regulation and epithelial mesenchymal transitions in breast cancer progression. *Journal of Mammary Gland Biology and Neoplasia*.

[45] Sánchez-Tilló E., Liu Y., de Barrios O. (2012). EMT-activating transcription factors in cancer: beyond EMT and tumor invasiveness. *Cellular and Molecular Life Sciences*.

[46] Tanaka Y., Terai Y., Kawaguchi H. (2014). Prognostic impact of EMT (epithelial-mesenchymal-transition)-related protein expression in endometrial cancer. *Cancer Biology & Therapy*.

[47] Gonzalez D. M., Medici D. (2014). Signaling mechanisms of the epithelial-mesenchymal transition. *Sci Signal*.

[48] Bolos V., Peinado H., Perez-Moreno M. A., Fraga M. F., Esteller M., Cano A. (2002). The transcription factor Slug represses E-cadherin expression and induces epithelial to mesenchymal transitions: a comparison with Snail and E47 repressors. *Journal of Cell Science*.

[49] Gupta P. B., Kuperwasser C., Brunet J. P. (2005). The melanocyte differentiation program predisposes to metastasis after neoplastic transformation. *Nature Genetics*.

[50] Campo L., Zhang C., Breuer E. K. (2015). EMT-Inducing Molecular Factors in Gynecological Cancers. *BioMed Research International*.

[51] Dhasarathy A., Kajita M., Wade P. A. (2007). The transcription factor snail mediates epithelial to mesenchymal transitions by repression of estrogen receptor-alpha. *Molecular Endocrinology*.

[52] Ye Y., Xiao Y., Wang W. (2010). ERalpha signaling through slug regulates E-cadherin and EMT. *Oncogene*.

[53] Zhang H., Li H., Qi S. (2016). Normal endometrial stromal cells regulate 17*β*-estradiol-induced epithelial-mesenchymal transition via SLUG and E-cadherin in endometrial adenocarcinoma cells in vitro. *Gynecological Endocrinology*.

[54] Huszar M., Pfeifer M., Schirmer U. (2010). Up-regulation of L1CAM is linked to loss of hormone receptors and E-cadherin in aggressive subtypes of endometrial carcinomas. *The Journal of Pathology*.

[55] Montserrat N., Mozos A., Llobet D. (2012). Epithelial to mesenchymal transition in early stage endometrioid endometrial carcinoma. *Human Pathology*.

[56] Storci G., Sansone P., Trere D. (2008). The basal-like breast carcinoma phenotype is regulated by SLUG gene expression. *The Journal of Pathology*.

[57] Yao C., Su L., Shan J. (2016). IGF/STAT3/NANOG/slug signaling axis simultaneously controls epithelial-mesenchymal transition and stemness maintenance in colorectal cancer. *Stem Cells*.

[58] Wu W. S., Heinrichs S., Xu D. (2005). Slug antagonizes p53- mediated apoptosis of hematopoietic progenitors by repressing puma. *Cell*.

[59] Singh A., Settleman J. (2010). EMT, cancer stem cells and drug resistance: an emerging axis of evil in the war on cancer. *Oncogene*.

[60] Liu Y., Nan F., Lu K. (2017). Identification of key genes in endometrioid endometrial adenocarcinoma via TCGA database. *Cancer Biomarkers*.

[61] Alonso-Alconada L., Muinelo-Romay L., Madissoo K. (2014). Molecular profiling of circulating tumor cells links plasticity to the metastatic process in endometrial cancer. *Molecular Cancer*.

[62] Creutzberg C. L., van Putten W. L., Koper P. C. (2000). Surgery and postoperative radiotherapy versus surgery alone for patients with stage-1 endometrial carcinoma: multicentre randomised trial. *Lancet*.

[63] Kosary C. L. (1994). FIGO stage, histology, histologic grade, age and race as prognostic factors in determining survival for cancers of the female gynecological system: an analysis of 1973–87 SEER cases of cancers of the endometrium, cervix, ovary, vulva, and vagina. *Seminars in Surgical Oncology*.

[64] Lindauer J., Fowler J. M., Manolitsas T. P. (2003). Is there a prognostic difference between depth of myometrial invasion and the tumor-free distance from the uterine serosa in endometrial cancer?. *Gynecologic Oncology*.

